# Recent advances in the understanding of Dupuytren’s disease

**DOI:** 10.12688/f1000research.17779.1

**Published:** 2019-02-28

**Authors:** Thomas Layton, Jagdeep Nanchahal

**Affiliations:** 1 The Kennedy Institute of Rheumatology, Nuffield Department of Orthopaedics, Rheumatology and Musculoskeletal Sciences, University of Oxford, Oxford, UK

**Keywords:** Dupuytren's disease, pathogenesis, localised inflammation, surgery, outcome measures

## Abstract

Dupuytren’s disease (DD) is a common fibrotic disorder of the hand and can significantly impair hand function. Although the exact pathogenesis of this disorder remains to be elucidated, immunological, genetic and cellular factors likely interact. In this review, we summarise recent advances in the understanding of DD pathogenesis and look to the future for potential novel therapeutic targets. In addition, we discuss the therapeutic options in DD with a focus on the need for more rigorous evidence to allow a meaningful comparison of different treatment modalities.

## Introduction

Dupuytren’s disease (DD) is a common and progressive fibroproliferative disorder of the palmar and digital fascia of the hand and, in Western populations, affects 12% among those who are 55 years old, increasing to 29% of those who are 75 years old ^1^. The initial clinical presentation is the appearance in the hand of a firm nodule, which expands into fibrous collagenous cords that extend into the digits. As the disease progresses, the cords mature, thicken and contract, leading to permanent flexion deformities. Disease progression is variable, but around 20 to 40% of patients eventually develop some degree of flexion deformity that can impair hand function ^2– 6^. Indeed, this deformity can significantly limit activities of daily living, including self-care and employment, and reduce health-related quality of life ^7^.

The current guidelines for the management of DD recommend intervention when the digital flexion contractures limit hand function and the proximal interphalangeal ^3, 8– 11^ or the metacarpophalangeal joint are flexed to 30° or more ^3, 8– 11^. A wide range of treatment options for late-stage disease are available, ranging from division of the cords using needle fasciotomy or collagenase injection through to surgical excision of the diseased tissue by limited fasciectomy or dermofasciectomy. Over recent years, there has been a determined effort to improve the evidence base to inform the management of DD, but much remains to be done. This article reviews recent advances in DD with an emphasis on the pathophysiology of the disease and current and emerging therapeutic paradigms.

## Emerging concepts in the pathogenesis of Dupuytren’s disease

### Genomics of Dupuytren’s disease

Although the aetiology of DD remains unknown, genetic, immunological and environmental factors likely interact to promote the development of this disease. It is well established that DD has a substantial heritable component, and a twin study from Denmark estimated the overall heritability as 80% ^12^. Despite this, only recently have we begun to uncover the genetic basis of the disease. A genome-wide association study (GWAS) identified nine susceptibility genetic loci in DD, six of which harboured genes encoding proteins in the Wnt signalling pathway, including *WNT4*, *SRFP4* and *RSPO2* ^13^. Wnt signalling has been associated with many other fibrotic diseases ^14^ but this was the first study highlighting this pathway as a potential key pathogenic driver in DD. Since then a number of studies have reported activation of Wnt signalling in DD ^15^, including increased expression of the Wnt signalling protein Wnt7b in nodules ^16^.

More recently, the largest GWAS in DD to date almost tripled the known risk loci, bringing the total known predisposing variants to 26 ^17^. This more recent study confirmed the association between DD and Wnt signalling, and many genes adjacent to risk loci are involved in this pathway. One intriguing novel finding from this study was decreased secretion of SFPR4, a soluble Wnt antagonist, in individuals homozygous for one high-risk allele. In other organs, downregulation of SFPR4 has been shown to promote myofibroblast activation during fibrosis ^18, 19^. These results provide an elegant link between a genetic predisposition to fibrosis in DD and activation of Wnt signalling mediated through *SFPR4* ^13, 16, 17^. Our understanding of the precise role of the genome in the pathogenesis of DD remains incomplete, but it appears that Wnt signalling may be crucial. Future work focused on how perturbations in this pathway influence stromal cell phenotypes may help to identify novel therapeutic targets.

### The immunology of Dupuytren’s disease

The development of fibrosis is almost invariably associated with persistent low-grade inflammation ^20, 21^, and various immune cells have been implicated in the pathogenesis of DD, including macrophages ^22, 23^, lymphocytes ^24^ and dendritic cells ^25^. It is only over the past few years that we have begun to appreciate the full complexity of the immune cell compartment, but the precise mechanism of immune cell activation is not understood ^26^. About 10% of the cells in DD nodules comprise immune cells, and the major populations are macrophages and lymphocytes. These cells secrete a diverse array of pro-inflammatory cytokines, including tumour necrosis factor (TNF), interleukin-6 (IL-6) and IL-8 ^22^. Although DD may be considered a localized inflammatory disorder, the exact mechanism of immune cell recruitment remains to be discovered. It has been suggested that DD is a T cell–mediated autoimmune disorder, largely based on the discovery of dense T-cell infiltrates in DD nodules ^24^. Other studies have confirmed the presence of different T-cell populations in nodules, including T helper cells ^27^. A recent study showed the presence of an activated T-cell infiltrate adjacent to blood vessels within the nodules and these cells expressed a restricted T-cell receptor repertoire *in vitro*. Despite the experimental limitations of this study, the findings support the concept that the local immune reaction may be triggered against an autoantigen, potentially secondary to microvascular changes within the hand ^26^. However, conclusive evidence for an adaptive immune response is still lacking and no autoantigen has been discovered or validated. Given the lack of validated animal models for DD and the heterogeneity in disease progression, uncovering the trigger for immune infiltration may be challenging. Even so, localized inflammation, regardless of its stimulus, is established in DD nodules and is likely key in driving myofibroblast activation and disease chronicity.

Various cytokines and growth factors have been associated with DD, including transforming growth factor-beta 1 (TGF-β1), platelet-derived growth factor (PDGF), TNF and IL-1β ^28^. Cells from DD nodules secrete a diverse array of soluble mediators, and the three most prominent cytokines are TGF-β1, TNF and IL-6 ^22^. The pleiotropic cytokine TNF has emerged as a key driver of the myofibroblast phenotype in DD and a novel therapeutic target. TNF was shown to selectively upregulate pro-fibrotic genes ( *COL1A1* and *ACTA2*) and proteins in palmar dermal fibroblasts from patients with DD, but not in non-palmar cells from the same patients or palmar cells from normal individuals ^22^. Importantly, the authors showed that the TNF acted via the Wnt signalling pathway by inhibiting GSK3β ^22^. *In vitro*, neutralizing antibodies to TNF resulted in downregulation of myofibroblast contractility in a dose-dependent manner ^22^. These data formed the foundation for a phase 2a placebo-controlled dose-ranging trial of anti-TNF (adalimumab) ^29, 30^. Direct intranodular injection of 40 mg of adalimumab in a concentrated formulation (0.4 mL) resulted in downregulation of procollagen type 1 and alpha-smooth muscle actin (α-SMA) proteins at 2 weeks post-injection ^30^. An ongoing phase 2b trial is comparing intranodular adalimumab with placebo in patients with early-stage DD ^29^. If the trial is successful, this will represent the first targeted treatment to control the progression of early-stage disease.

### The extracellular matrix in Dupuytren’s disease

The extracellular matrix (ECM) is one of the most important regulators of cellular and tissue function ^31^, and fibrosis results when ECM homeostasis is lost. Much is known about the matrix composition in DD and this differs according to the stage of the disease ^32– 34^. Cords are composed of mature fibrillar collagen, with relatively few cells ^32, 33, 35^, which mainly comprise fibroblasts ^33^. Nodules, in contrast, are highly cellular with densely packed myofibroblasts ^22, 32, 34, 36^ in an irregular pallisadal pattern. Despite the plethora of descriptive studies on both of these structures, we still have a poor understanding of their origin and lack definitive evidence that one leads to the other, although indirect evidence points to nodules as the precursor of late-stage cords ^32, 33^. Nodules are enriched with immune and proliferating cells and likely represent the active disease unit ^32^. One proposed model describes the cells within the nodules first secreting and contracting the matrix component ^33^. This is then remodelled and maintained as a cord. As the fixed flexion deformity develops, myofibroblast numbers decrease, perhaps through apoptosis, and the cords become less cellular ^32, 33^. Regardless, formation and shortening of the cord by the myofibroblasts are necessary for the development of flexion contractures that ultimately compromise hand function ^33, 37^.

The significance of ECM homeostasis in DD is highlighted by several single-nucleotide polymorphisms in GWASs associated with matrix remodelling, including discoid domain receptor ( *DDR2*), matrix metalloprotease 14 ( *MMP14*) and integrin alpha-11 ( *ITGA11*) ^17^. These molecules are attractive candidates for promoting fibrosis in DD and function to bind various matrix proteins and regulate their turnover. DDR2 is a membrane receptor tyrosine kinase whose ligands include type I and III fibrillar collagen, the most prominent ECM proteins found in DD nodules and cords ^32, 34, 35^. DDR2 has been shown to regulate fibrosis in the lung and liver but its exact function in DD remains elusive ^38– 40^. In lung fibroblasts, DDR2 synergized and potentiated the action of TGF-β1 and fibrillar collagen in stimulating myofibroblast differentiation ^40^. It is likely that in DD a similar mechanism promotes the activation of fibroblasts and induces collagen deposition in the palmar fascia. *MMP14* (MT1-MMP) is another gene associated with a high-risk locus and is a type I transmembrane protein of the MMP family of proteases. MMP14 is overexpressed in DD nodules, and broad-spectrum inhibition of MMPs in clinical trials for cancer led to some individuals developing DD ^41^. These data point to a potential for this protein as a crucial regulator of fibrosis in DD. Supporting this, knockdown of *MMP14* in DD fibroblasts inhibited both cell contraction and MMP2 activation *in vitro* ^42^. Further work is warranted to investigate how MMP14 regulates the myofibroblast phenotype and may validate this protein as a therapeutic target in DD. Interestingly, work has demonstrated that MMP gene expression correlates with clinical outcomes, including recurrence following treatment ^43^.

Mechanical forces are vital in promoting the development and progression of fibrosis ^44^. These forces regulate myofibroblast function in addition to the structure and mechanics of the matrix ^45, 46^. Generally, as fibrosis progresses, the matrix protein deposition leads to tissues becoming stiffer. In turn, this promotes further matrix secretion by myofibroblasts, thereby creating a positive feedback loop that maintains the disease pathogenesis. Given the importance of tissue mechanics in fibrosis and the potential for its modulation as a therapeutic, much work has been undertaken to elucidate the pathways governing cellular mechanotransduction. The key mechanotransduction molecules that have emerged are the transcriptional co-activators Yes-associated protein 1 (YAP1) and transcriptional co-activator with PDZ-binding motif (TAZ) ^47^. These are members of the Hippo pathway and have been shown to regulate global changes in gene expression in response to tissue stiffness ^47^. In addition, YAP1 has been demonstrated to regulate the myofibroblast phenotype and transduce signals from the mechanical environment in several fibrotic conditions ^44, 48^. Moreover, in an *in vitro* fibroblast spheroid system, TAZ activation resulted in increased cell contraction and ECM expression in response to increasing stiffness ^49^. More recently, YAP1 has been shown to be a crucial determinant of the myofibroblast phenotype in DD. Silencing of *YAP1* in DD myofibroblasts demonstrated its key role in the expression of fibrotic genes and cell contraction ^48^. Whether YAP1 and TAZ are attractive therapeutic targets in DD remains to be confirmed, but collectively emerging work has highlighted the potential for targeting mechanosensitive pathways.

## Emerging and existing treatments for Dupuytren’s disease

### Non-surgical

There is no definitive cure for DD, and current treatments for late-stage disease aim to correct the flexion deformity of the finger and restore hand function. Although the mainstay of treatment for patients with established flexion deformity is surgery, excision of the more cellular, proliferative stage of the disease is considered to be associated with a higher rate of recurrence ^50^. Numerous non-surgical treatments, including pharmacological treatment with vitamin E or steroids, physical therapies and radiotherapy, have been described for earlier-stage disease ^51^. Despite the plethora of publications, descriptions are limited to uncontrolled and unblinded studies and there is no conclusive evidence for their efficacy ^51^.

The pursuit of a more minimal treatment in DD now also encompasses late-stage disease. Collagenase histolyticum (CCH) injections ^52^ benefit from being less invasive than surgery with more rapid recovery and can be performed in the office, and complications are transient ^53– 55^. CCH enzymatically disrupts the cord, and more widespread use has galvanized recent efforts to develop a more robust evidence base for its role ^52, 56– 60^. Although CCH has been shown to reduce joint contracture and improve the range of joint motion compared with placebo and appears as efficacious as percutaneous needle fasciotomy (PNF), it may not be cost-effective, at least in the US ^61, 62^. Ongoing multi-centre clinical trials comparing the efficacy and cost-effectiveness of CCH with surgical excision should help to definitely address some of these issues ^63^.

### Surgery in Dupuytren’s disease

The mainstay of treatment for late-stage DD remains surgery, and several operative procedures are available ^64^. These include needle fasciotomy (aponeurotomy), limited fasciectomy and dermofasciectomy. These techniques vary in their invasiveness and have their own advantages and limitations. Generally, more invasive procedures are associated with lower risk of recurrence but necessitate longer post-operative rehabilitation ^65– 68^. In PNF, the cords are divided with a hypodermic needle. The advantage of this technique is that it is less invasive than fasciectomy and may be used in the outpatient setting. A number of studies have demonstrated improvement in flexion deformities with PNF, but with a relatively high risk of recurrence of about 30% at 5 years compared with 6% for limited fasciectomy ^69^. A randomized controlled trial of PNF demonstrated efficacy comparable to that of CCH injection for correction of flexion contraction deformity but with the potential for a higher risk of recurrence ^56, 70^.

Limited fasciectomy involves excision of the majority of the diseased tissue whilst preserving the overlying palmar skin. In dermofasciectomy, the excision is extended to include all subcutaneous fat and skin overlying the diseased tissue and necessitates the use of a full-thickness skin graft ^65^. The proposed advantage of these techniques is reduced risk of recurrence as compared with minimally invasive procedures such as PNF, together with better correction of flexion deformity ^65, 69, 71^. A potential benefit of dermofasciectomy is more radical clearance of the diseased tissue as well as potential myofibroblast precursors in the overlying fat and dermis, and a multi-centre cross-sectional study reported the reoperation rate following fasciectomy as 6% compared with 0% after dermofasciectomy at 5 years ^72^. However, residual impairment of hand function did not differ between procedures, even when reoperation and other variables were controlled. One potential reason for this is the long-term morbidity associated with the higher post-operative complications in dermofasciectomy. Dermofasciectomy remains a highly variable procedure and there are large differences in the size of skin graft used ^65^. It is important that future comparative studies evaluate hand function rather than simply relying on the surgeon’s assessment of recurrence ^73^ or angular measurement of digital deformities. This in turn requires well-defined outcome measures.

### Outcomes in Dupuytren’s disease

Outcome measures that robustly assess the function of the hand and impact across a range of daily activities in patients with disparate demands, whilst being sensitive to change following treatment across the timescales considered, are essential to ascertain the efficacy of any intervention. Even with objective measures, consensus is only just beginning to emerge ^74^. However, these measures often fail to capture the patient’s perspective on their treatment success with the potential for profound bias in the results ^74^. For example, if an intervention successfully corrects the angular deformity of the finger but leaves the patient with cold intolerance or chronic regional pain syndrome, it may still have failed to achieve meaningful clinical improvement. The use of patient-reported outcomes measures (PROMs) aims to bridge this gap and empower the patients during evaluation of their treatment. A number of PROMs exist for DD, and the most common are the Disabilities of the Arm, Shoulder and Hand (DASH) ^75^ or disease-specific instruments such as the Unité Rhumatologique des Affections de la Main (URAM) ^76, 77^. Theses scales aim to assess the impact on the patient’s quality of life and include questions on washing, daily tasks and recreational activities. Nonetheless, recent studies have demonstrated limitations of using DASH for DD, highlighting the context dependence of these measures ^78, 79^. Ultimately, a combination of measures such as region-specific questionnaires such as the Michigan Hand Questionnaire, disease-specific instruments like the URAM, and measures of patient satisfaction combined with assessment of range of motion, grip and sensibility may be required to enable meaningful comparison of different treatment modalities for DD ^76^.

## Conclusions

Although DD remains a significant burden in the Western world, we are only just beginning to understand its pathogenesis. Alongside growing recognition of the molecular mechanisms driving DD, there is an increasing awareness for the necessity to evaluate the efficacy of current surgical and non-surgical interventions. Recent work has emphasized the need to improve our evidence base for the management of DD, including gaining consensus for robust outcome measures that reflect function and include the patient’s perspective. Achieving these goals will provide a strong platform to define novel therapeutic targets and treatment paradigms to optimise patient care.
